# Optimal design of water reuse networks in cities through decision support tool development and testing

**DOI:** 10.1038/s41545-023-00222-4

**Published:** 2023-03-17

**Authors:** Eusebi Calle, David Martínez, Gianluigi Buttiglieri, Lluís Corominas, Miquel Farreras, Joan Saló-Grau, Pere Vilà, Josep Pueyo-Ros, Joaquim Comas

**Affiliations:** 1 Institute of Informatics and Applications, University of Girona, Girona, Spain; 2 Catalan Institute for Water Research (ICRA-CERCA), Emili Grahit 101, 17003 Girona, Spain; 3 University of Girona, Girona, Spain; 4 LEQUIA, Institute of Environment, University of Girona, E-17071 Girona, Spain

**Keywords:** Environmental sciences, Sustainability

## Abstract

Water scarcity and droughts are an increasing issue in many parts of the world. In the context of urban water systems, the transition to circularity may imply wastewater treatment and reuse. Planning and assessment of water reuse projects require decision-makers evaluating the cost and benefits of alternative scenarios. Manual or semi-automatic approaches are still common practice for planning both drinking and reclaimed water distribution networks. This work illustrates a decision support tool that, based on open data sources and graph theory coupled to greedy optimization algorithms, is able to automatically compute the optimal reclaimed water network for a given scenario. The tool provides not only the maximum amount of served reclaimed water per unit of invested cost, but also the length and diameters of the pipes required, the location and size of storage tanks, the population served, and the construction costs, i.e., everything under the same architecture. The usefulness of the tool is illustrated in two different but complementary cities in terms of size, density, and topography. The construction cost of the optimal water reclaimed network for a city of approximately 100,000 inhabitants is estimated to be in the range of €0.17–0.22/m^3^ (for a payback period of 30 years).

## Introduction

Water resources are limited and unequally distributed in space and time. Water scarcity and droughts are an increasing problem in many areas, at least seasonally, in terms of intensity and frequency^1^. Tourism has been recognized as one of the most significant water-consuming sectors on local, regional, and global scales^2,3^, as its viability and sustainability depends on adequate water supply quantity and quality^4^.

In the context of urban water systems, the transition to circularity and minimizing potable water consumption, requires the redesign of the water infrastructure, including (waste)water treatment and water reuse^5^. Treated wastewater can be used for non-potable purposes, including irrigation, toilet flushing, car washing, cleaning purposes, and industrial uses^6^, where appropriate technologies should be carefully selected. EU legislation (EU 2020/741) sets minimum requirements, especially for agricultural water reuse purposes. It does, however, not specifically regulate water reuse in tourist facilities or water reuse for general urban uses, such as toilet flushing. Spain is one of the only five European countries, besides Cyprus, Greece, France, and Italy, which have implemented a national legally binding water reuse regulation (RD 1620/2007). The Spanish water reuse regulation, in fact, is currently the regulation in the EU with the highest number of well-defined water reuse applications, including toilet flushing and garden irrigation. Beyond Europe, other countries worldwide, such as USA, Australia, Singapore, and South Africa, also allow using reclaimed water in cities and specifically for domestic uses. Besides, it is expected that more and more countries will soon consider water reuse as a reliable alternative resource. Several municipalities in Spain (e.g., Sant Cugat del Vallès) have been promoting water reuse in multi-story buildings^7,8^. Nonetheless, applications are still very limited, and related information is largely lacking in the literature.

Efficient and sustainable water reuse requires feasible water reuse projects (i.e., water reclamation treatment plants and distribution to potential uses). Planning and assessing water reuse projects require decision-makers answering a number of questions concerning issues such as: (i) the best tertiary/advanced treatment to be implemented, (ii) the number of uses/users in the city (i.e., how much wastewater needs to be reclaimed), and (iii) how to select the optimal water distribution network. The answer to these questions requires considering different challenges (environmental, economic) and technologies for water reclamation and potential uses of reclaimed water, while evaluating the cost and benefit of all the scenarios by means of different criteria.

In a seminal work^9^, a life cycle assessment study was carried out to evaluate the impact of water reuse in the city of Lloret de Mar (Catalonia, NE of Spain), a mass tourism destination on the Mediterranean coast with a high density of high-rise hotels. This study considered four distinct scenarios: non optimized (only potable water consumption), decentralized, hybrid, and centralized. All the water distribution networks were designed manually, assuming the shortest path with lowest terrain elevation. In fact, the multiple factors that need to be considered (terrain elevation, street graph, pipe diameters, terrain usages, etc.) mean that manual or semi-automatic design is still the common practice in planning distribution networks, both for drinking and reclaimed water.

Multiple data, knowledge from different disciplines, and computational capabilities need to be integrated. This is a complex problem where model-based and decision support tools can help by offering a variety of solutions. Previous research on decision support tools for wastewater management has been mainly focused on wastewater treatment^10,11^, with a few recent examples dealing with the selection of the most adequate advanced treatment technologies for water reclamation^12,13^. The design of reclaimed water distribution networks has attracted little attention.

The problem of planning and identifying the most suitable economic schemes for centralized wastewater infrastructure has been partially solved^14^. This solution is based on the potential of a geographical information system to design and locate the water collection pipe network. However, it only considers on-site water reuse, i.e. no water reclamation networks are required. In addition, an improved data-reduced method for wastewater management using globally available data has been proposed^15^ (i.e., GIS and statistical data), enabling the approach to be applied worldwide. On the other hand^16^, del Teso et al*.* (2019) aims at energy optimisation in drinking water distribution networks, considering not only operational losses but also structural (or topographic) ones. However, none take into account the initial (or brand-new) design of water reclamation networks. Moreover, more than one tool is sometimes used in a sequential manner, with the corresponding conversion of variables and parameters between the tools, thus becoming highly time-consuming work, especially when aiming at optimal designed networks^17^.

In^18^, the authors evaluate the life cycle costs and benefits of decentralized greywater reuse planning based on two scales of decentralization: satellite and onsite. However, these two decentralization scales require separating raw greywater from wastewater at the source, which is often not possible in many cities. A centralized water reclamation plant and the corresponding water distribution network is not considered. There is also literature on urban stormwater management. The work of Khurelbaatar et al*.* (2021)^17^ shows an approach that uses the software package MIKE URBAN from DHI (MIKE URBAN, Hørsholm, Danmark) for estimating the potential for managing urban stormwater in already existing urban environments to mitigate the impact of urban stormwater runoff. However, few of their proposed scenarios allow for stormwater reuse.

Distribution water and wastewater network modeling can be approached through graph theory^19^, such as^20^, for designing water distribution networks based on loops hydraulically balanced method, and wastewater sensor placement approaches for SARS-CoV-2 detection^21^, but has not been used yet for the advanced and automated design optimization of water networks.

Given this background, the aim of this paper is to describe a decision support tool for planning water reuse networks in cities. Our approach integrates several algorithms for designing water reuse networks based on graph theory coupled to existent greedy optimization algorithms^22,23^. Our proposal is made up of one single tool, in contrast with the literature, avoiding the need for data exchange and thus resulting in potential savings in time and effort. This tool combines city characteristics (i.e., terrain characteristics, including plot and building usages, elevation, and slope) and water consumption rates to automatically propose an optimal network for water reuse. This paper proposes advanced algorithms to design and optimize large-scale water reuse networks. The usefulness of our solution is also illustrated when testing in real cities. And in this line, two cities of different scales and significantly diverse water uses and requirements have been compared. Construction costs and benefits in terms of water savings are estimated for each scenario. Finally, the optimal water network can be provided when only a limited budget is available.

## Results

### Overview

The section first describes the innovative decision support tool, called REWATnet, for planning water reuse projects, and highlights the steps involved and the outputs produced. Next, the application of the tool to generate and analyze potential water reuse projects for the cities of Girona and Lloret de Mar is presented. The location of water reclamation treatment plants for reclaimed water production in each city is considered the same as the existing wastewater treatment plants in centralized scenarios, assuming they include the necessary water treatments for the desired reclaimed water quality. The reclaimed water networks obtained by using different algorithms and different scenarios are compared, and the usefulness of the REWATnet tool for network optimisation, when a limited budget is available, is also illustrated and compared with a semi-manual approach.

### Description of the REWATnet decision support tool

**Fig. 1:**
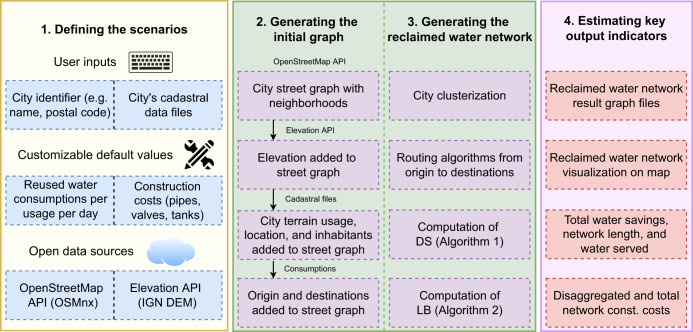
REWATnet decision support tool. REWATnet simplified scheme. The data flow is as follows: (i) defining the scenarios, including user inputs, customizable values, and open data sources; (ii) generating the initial graph, where an initial city street graph is generated from the OpenStreetMap API, the elevations are added to each node from the Elevation API, and the cadastral file is processed where consumptions are added to nodes; (iii) generating the reclaimed water network, which includes the application of the algorithms such as city clusterization, routing algorithms, pipe diameter selection (DS) and limited budget (LB), to the initial graph; and (iv) estimating key output indicators, where the result graph files with the reclaimed water network, visualizations on map, water saving, network length, water served, and disaggregated and total network construction costs are obtained.

The new REWATnet decision support tool for planning urban water reuse projects involves the following steps: (i) defining the scenarios; (ii) generating the initial graph; (iii) generating the reclaimed water network; and (iv) estimating key output indicators. The tool provides an innovative mechanism for designing reclaimed water networks in Spain, which can be easily adapted to any country. Figure 1 illustrates a simplified scheme of the water reuse planning tool.

#### Defining the scenarios

Defining the scenarios to be simulated consists of defining the user inputs, gathering open source data and, only when necessary, customizing default values. The only required user inputs are the target city identifier, the city’s cadastral data files, and the location of the wastewater treatment plant. On the one hand, the city identifier is used to obtain the city’s street graph through the OpenStreetMap API (see “Methods” section), and must be specified by name, postal code or OpenStreetMap identifier. On the other hand, the same city identifier is also used to gather the city’s topography Digital Elevation Model (DEM), which is essential to obtain the city street graph nodes elevation (see “Methods” section). The elevation data can be commonly obtained from the country’s official geographic data provider: In Spain this is the Instituto Geográfico Nacional^24^. The DEM is obtained from this source with a 5x5 meter precision.

The city’s cadastral data files are used to gather land plot and building data. In Spain, land plot and building data can be obtained from the official cadastre online database. This data is divided into four files that can be freely downloaded: two “.cat” extension files containing the urban and rural plots and buildings, and two “.shp” extension files containing the public gardens in both urban and rural zones. As these files are presented in a hard-to-process format, and for faster and simpler data treatment, said files are converted and combined to a unique standard JSON (JavaScript Object Notation^25^) file using a Python^26^ custom implemented script. For other countries, it would be necessary to process the corresponding authority land plot data and generate the standard JSON file. In the case of several municipalities connected to the same reclamation water treatment plant, the files from every municipality must be downloaded, converted to JSON with the script, and merged to form a unique file. Additionally, the OverPass API^27^ and Shapely^28^ library allow for the location and surface of public gardens to be obtained and appended since they are not included in cadastre files.

Optional user inputs are the definition of a subset of the considered uses for reclaimed water, a threshold for minimal water consumption and specific city areas particularly suitable for water reuse. The subset of considered uses for reclaimed water will define the network destinations. The threshold for water consumption may specify whether a minimum amount of reclaimed water must be served in each network destination. The selection of specific city areas especially suitable for water reuse may be relevant to target new city settlements (i.e., neighborhoods or sectors) and for statistical and future work purposes. The OverPass API is used to extract specific city areas (downloading the geographic polygons that define the divisions) together with the Shapely library (checking to which polygon a certain node belongs). By default, all water use categories, a threshold for water consumption of 0 m^3^/d for all network destinations (see Methods section) and the whole city area are considered for water reuse projects.

#### Generating the initial graph and the reclaimed water network

Once the scenario is defined and all data has been gathered, the REWATnet decision support tool is able to generate the reclaimed water network. First, an initial graph is generated and then the different optimization algorithms are applied (see Methods section).

The initial graph is generated by adding the data to the city street graph, including the node elevation, street slopes, city terrain usage, location, and inhabitants, as well as the origin node and the destination nodes. Then, the routing algorithms are computed from the initial graph to obtain the optimal reclaimed water network based on two approaches: (i) serving water to the destinations defined in the scenarios whatever the budget might be, or (ii) maximizing the water served with a limited budget. In the first approach, city clustering algorithms may be first applied to the initial graph depending on the targeted city size. Then, the routing algorithms are computed for the main network (i.e., from the initial node (water tank next to the water treatment plant) to the cluster water tanks distributed along the city) and the branched network areas (i.e., from each cluster water tank to all its destinations). The routing algorithms generate the base for the reclaimed water network, although the network construction costs remain unknown as the pipe diameters have not yet been computed. Then, the computation of the pipe diameter selection (DS) algorithm (Algorithm 1) generates all the pipe diameters of the reclaimed water network and enables the construction costs to be calculated (see Supplementary Table 1, Supplementary Table 2, and Supplementary Table 3).

In the second approach, the Limited Budget (LB) availability algorithm (Algorithm 2) is computed considering a single branched network area. The LB algorithm uses the routing algorithms and the DS algorithm to build a reclaimed water network that maximizes the water volume served for a specific budget (see Methods section).

#### Estimating key output indicators

The REWATnet decision support tool estimates the following key output indicators:

- Graph files of the optimal reclaimed water network: the optimal reclaimed water network is provided in a file with a standard graphml format for further analysis.
- Visualization on a map of the optimal reclaimed water network: a clear visual representation of the reclaimed water network drawn on a plane and over a map, provided in PDF vector image files.
- Network length, pipe diameters, population served, and total water savings: this relevant data related to each reclaimed water network is provided in a simple text file. The total water savings is assumed as the total reclaimed water consumption, as this amount of water will be subtracted from the drinking water distribution network.
- Disaggregated and total network construction costs: the construction costs of the reclaimed water network are provided disaggregated by main network costs, branched network costs, and water tank costs expressed in thousands of euros (€K), provided in another simple text file.

Before the execution and analysis of the different scenarios, a preliminary validation of the REWATnet tool was carried out for the case study of Girona. The reclaimed water consumption of potential users estimated by the tool was compared to actual water consumption data provided by the water authorities (see Table 1). The estimated model consumption was calculated based on the methods and references of Supplementary Table 4 in Supplementary Materials, together with the information extracted from the land plots (see “Methods” section). The validation shows a remarkably accurate model consumption with a minor overall error of 6.4%. According to the water use categories, the error between actual and estimated consumption is especially low for both economic operations and domestic use categories, as shown in Table 1. In the case of the public uses, which suppose the lowest water consumption, the model shows an error of 23.8%. This error barely affects the overall error of 6.4% as it represents only 10% of the total consumption. The model estimates a higher public uses consumption as the toilet flushing and irrigation consumption data in sports centers is complicated to estimate, and the land plot data may be incomplete or outdated. Finally, it is worth noting that the consumption for vegetable garden irrigation has not been included since there is no real data to be compared with. It should also be noted that when validation results are not satisfactory (which is not the case of the present work), the tool allows for the fine-tuning of some parameter values of the estimated consumption rates (Table 1).

**Table 1:** Water use consumption validation (Girona).

Water use categories	Model consumption (m^3^/year)	Actual consumption (m^3^/year)	Error
Economic operations (Domestic with operations + Large industrial consumption)	530,564	525,588	2.8%
Public uses (Urban equipment + Public garden irrigation)	251,405	203,064	23.8%
Domestic (Housing + Private garden irrigation)	1,690,877	1,594,735	6%
All validated uses* (Economic operations + Public uses + Domestic)	2,472,846	2,323,387	6.4%

*All validated uses do not include the vegetable garden irrigation model consumption (estimated as 269,005 m^3^/year) as there is no actual consumption data to be compared.

### Comparison of different routing algorithms

Among the Kou, Takahashi, and Mehlhorn routing algorithms, the Mehlhorn algorithm was found to be the most suitable in the literature due to its lower computational complexity (see “Methods” section). Nonetheless, all routing algorithms were tested with our case study cities to evaluate their accuracy, where lower reclaimed network length provides better accuracy. Thus, the planning tool has been first tested for designing the optimal reclaimed water network for the whole urban area of Girona and Lloret de Mar cities applying the Kou, Takahashi, and Mehlhorn algorithms. In both cities, reclaimed water is produced in a centralized water reclamation plant and stored in an initial water tank placed alongside it. The whole city of Lloret de Mar is considered as a unique cluster (i.e., one branched network area), while city clustering techniques are applied in the case of Girona to determine the optimal placement of water tanks. In fact, due to the city’s size, intermediate water tanks are needed along the reclaimed water network for the case of Girona. In both case studies, only public water uses are considered. Besides, we contemplate water reuse destinations within 300 m between any land plot centroid (i.e., the geographical center of the physical entity that uses reclaimed water) and the nearest node of the city’s initial street graph. This consideration is necessary to ignore water destinations obtained from land plots’ official data that are too far from the street graph (e.g., a farm outside the city), as the reclaimed water network is built based on the streets. Given this scenario, the initial graph of Girona considers 328 destinations with total water consumption of 2129 m^3^/d, while the initial graph of Lloret de Mar considers 144 destinations with total water consumption of 1182 m^3^/d.

Table 2 shows the comparison between the REWATnet output indicators for the different routing algorithms and the two cities. The first thing to notice is that, for both Girona and Lloret de Mar, the Kou and Mehlhorn algorithms present exactly the same accuracy (the same reclaimed network pipe length), while the Mehlhorn algorithm provides a significantly lower computation time (about 100 times faster). Although the Takahashi algorithm present the best accuracy (lower reclaimed network pipe length), compared to the Kou and Mehlhorn algorithms, its execution time becomes intractable on cities with a large set of destinations (about 18,025 and 117,179 times slower than the Mehlhorn algorithm).

**Table 2:** Comparison of the decision support tool output indicators for the different routing algorithms.

City	Algorithm	Network length (m)	Execution time (s)
Girona	Takahashi	67,149	28,123 (≈7.8 h)
	Kou	68,068	38.06
	Mehlhorn	68,068	0.24
Lloret de Mar	Takahashi	49,498	1442 (≈24 m)
	Kou	50,413	8.39
	Mehlhorn	50,413	0.08

### Comparison of reclaimed water networks

For both cities and using the best routing algorithm, the reclaimed water network was computed considering two scenarios serving water to all the destinations: (i) only for public water uses and (ii) for both public and private water uses. This section presents some of the most relevant key output indicators.

**Fig. 2:**
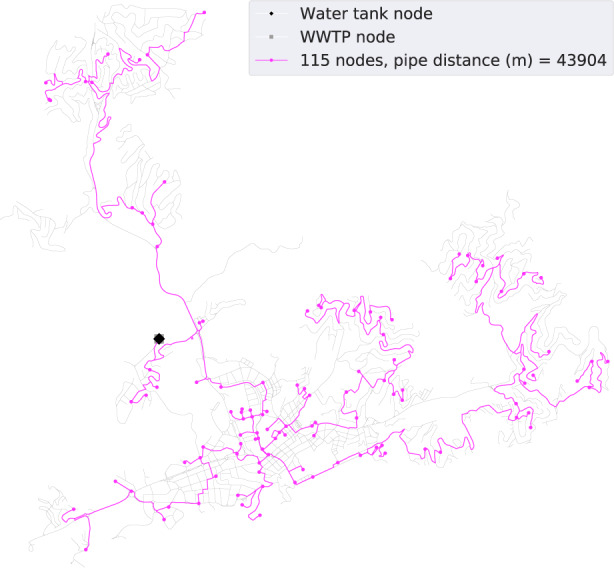
Reclaimed water network visualization (Lloret de Mar). The diagram on the left shows the resulting reclaimed water network for only public cases, while the one on the right shows the resulting reclaimed water network for both public and private uses. The city street graph is represented in gray paths and the reclaimed water network in magenta paths.

Regarding Lloret de Mar, Fig. 2 illustrates the graphs representing the reclaimed water networks generated for the two scenarios. The first scenario results in a 44 km network, a total construction cost of €3628K, a pipe diameter average of 64 mm, and total consumption of 283 m^3^/d to serve 4.1% of the total demanded water (Water served/total demand × 100). The second scenario results in a 104 km network, a total construction cost of €9429K, a pipe diameter average of 82 mm, and total consumption of 6844 m^3^/d to serve the entire demand for water. It is worth noting that the first scenario presents a construction cost per cubic meter of €12.82K/m^3^/d compared with the €1.38K/m^3^/d of the second scenario, which is more than nine times bigger. This difference might be related to the fact that private water use needs connections that are already (partially) included in the first scenario.

**Fig. 3:**
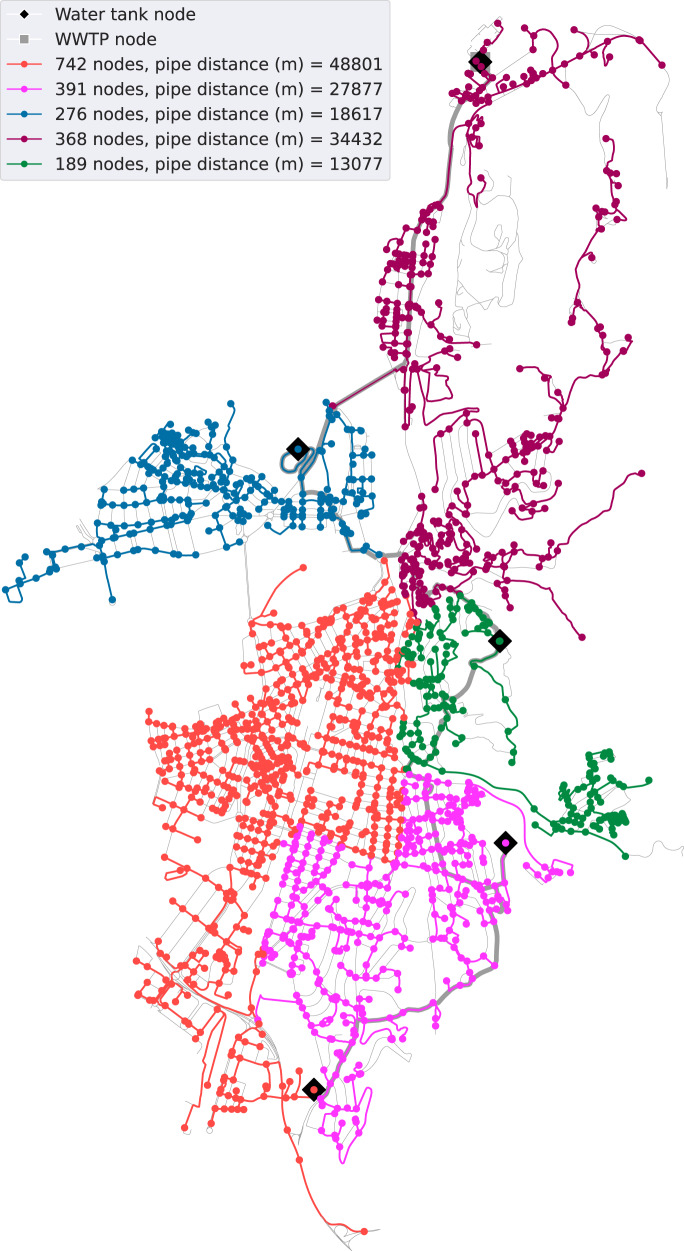
Reclaimed water network visualization (Girona). The reclaimed water network for the case study of Girona is illustrated with five clusters and for both public and private uses. The city street graph is represented in gray paths and the generated branched networks in different colors (for each cluster). Black rhombuses indicate the location of water tanks (origin point of a branched network). The main network is represented in wider gray paths.

Regarding Girona, Table 3 contains the disaggregated and total water reclaimed network construction costs obtained for different clustering solutions (from one to eight clusters) for the scenario (ii) of public and private water uses. The observed total water consumption is 7142 m^3^/d, leading to an average cost increase for each cluster of 4.2%. It is worth noting the low increase in the costs of the pipe network, as the branched network costs from two to eight clusters are analogous. Hence, the most significant elements that increase the cost among the different clustering solutions is the main network and the number of water tanks. Note that, the whole infrastructure payback period considered is 30 years, and the accumulative water savings should also be considered (up to 78,204,900 m^3^ of total consumption). The resulting reclaimed water network of five clusters is shown in Fig. 3, with 155 km of pipes and a total construction cost of €15,996K. Interestingly, in this case, the placement positions for the water tanks computed by the tool match well with the ones that are actually in the Girona drinking water distribution network^29^. In Supplementary Materials, the clusterization results of Girona with three (Supplementary Fig. 1) and seven (Supplementary Fig. 2) clusters are also illustrated.

**Table 3:** Reclaimed water network construction costs for different clustering solutions, both public and private uses (Girona).

Number of clusters	Main network costs (€K)	Branched network costs (€K)	Water tank costs (€K)	Total costs (€K)	Cost (€K/m^3^/d)
1	0	12,361	760	13,121	1.84
2	1177	11,278	1200	13,655	1.91
3	1421	11,213	1460	14,094	1.97
4	1715	11,300	1610	14,625	2.05
5	2533	11,502	1960	15,996	2.24
6	2812	11,198	2310	16,320	2.29
7	3249	11,254	2550	17,053	2.39
8	3305	11,291	2870	17,466	2.45

### Comparison of optimal network with current practice

The optimal network with limited budget availability is illustrated in the case study of Girona. The Limited Budget (LB) availability algorithm (Algorithm 2) results in the maximum amount of reclaimed water served given a maximum budget *B* (see Methods section). The LB algorithm is executed considering eight different budgets from €500K to €2000K, with intervals of €250K over a randomly selected branched network area, obtained from the previously generated five-cluster reclaimed water network (see Fig. 3). Table 4 shows these results for the case study of Girona where, considering the blue branched network area water tank as the origin (from the five cluster solution, see Fig. 3), each limited budget *B*, pipe network length, reclaimed water served, percentage of water served over total demand, and execution time output indicators are presented. The results show a linear evolution of the percentage of water served over total demand *C* as a function of budget *B*. Supplementary Figs. 3, 4, 5, 6, 7, 8, and 9 illustrate the networks generated for each budget (computation performed) in Table 4.

**Table 4:** LB algorithm results over a randomly selected branched network area (Girona five clusters, blue cluster water tank in Fig. 3, public and private uses).

Budget (€K)	Pipe network length (m)	Transported water (m^3^)	Water served / total demand (%)	Execution time (s)
500	3420	337	4.7	57
750	5191	529	7.4	59
1000	8464	777	10.9	159
1250	11,412	1228	17.2	273
1500	14,680	1477	20.7	384
1750	17,658	1906	26.7	392
2000	20,426	2086	29.2	493

**Fig. 4:**
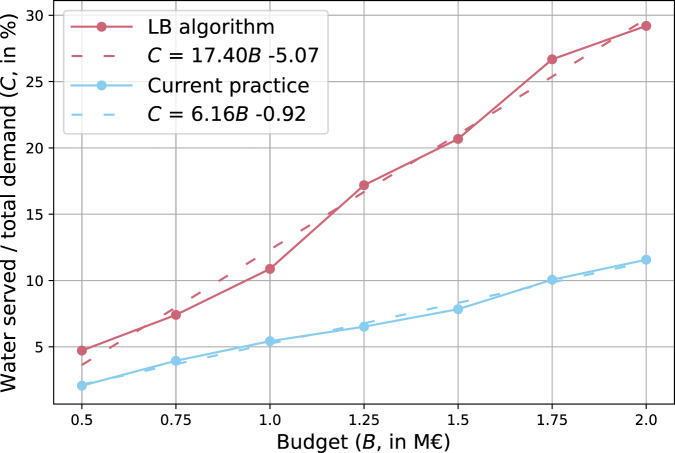
LB algorithm water served per budget results, compared with current practice. Percentage of water served over total demand comparison between the LB algorithm and current practice (manual approach). The linear regression functions for both the LB algorithm and current practice are defined in the figure legend and represented as dotted lines.

The LB algorithm optimization is compared with the so-named “current practice”, which considers a manual approach based on actual reclaimed water network planning experience. In the current practice, unlike the LB algorithm, the best profit *P* (water served per cost ratio) is considered based purely on the lowest distance without considering water served (i.e., for each iteration, the closest destination is added to the current reclaimed water network until budget *B* is reached). The current practice is applied with the same budgets considered in the testing of the LB algorithm, showing a linear evolution with a considerably lower slope of the percentage of water served over total demand *C* as a function of the budget. Both linear functions are illustrated in Fig. 4, where the LB algorithm results in a function *C* = 17.40*B*−5.07 and the current practice in a function *C* = 6.16*B*−0.92. As can be seen in Fig. 4, the benefits of the optimal network approach are more evident as the budget increases, since the slope of the LB algorithm is almost three times (2.82) that of the current practice.

## Discussion

In the context of the global climate change, where water scarcity regions are increasing and water reuse is becoming paramount, an optimal return (e.g. served water per unit cost invested) from each water reclamation project is sought. Moreover, tourism is recognized as a major water consuming sector, and the growth in tourism establishments has been matched by a growth in water demand^3^. As such, the new decision support tool presented has been applied to two case studies in Spain, but it can be easily adapted and applied to any region world wide. This would only require configuring the proper open online services and open data sources (e.g. cadaster) and a potential customization of default values for water consumption and costs. According to our knowledge, there are no similar tools in the literature able to plan and economically asses an optimal water reclamation network for a city with little computing effort. Besides, the tool may be used for usual water distribution networks, although it should be validated and revised.

Urban planners from municipal or regional authorities, consulting companies and/or water utilities can use the tool for planning urban water reuse projects, i.e., to identify a city’s most critical water consumption hotspots, compare different solutions by using technical and economic criteria and then select the optimal alternative. Another application for this innovative tool could be to assess which water reuse scenario - centralized, semi-decentralized, or decentralized - offers a better cost-efficient water reuse scheme, optimizing the number and location of decentralized treatment plants. Another question worth examining with the tool would be determining the minimum number of inhabitants to be decentralized for a water reuse solution to be sustainable for a specific city/neighborhood. REWATnet can also be used as a dissemination and training tool for planning water reuse schemes.

This decentralized water treatment and reuse can be extremely relevant for touristic cities, easing the pressure on scarce water resources and/or significantly reducing wastewater generation. For example, in Lloret de Mar, with 40,000 inhabitants in winter and up to 200,000 in summer, the portion of generated wastewater coming from tourist facilities was estimated as more than 10,000 m^3^/d and at least half of this amount was gray water, which can be more easily reclaimed than wastewater^30^.

A validation of the reclaimed network design (pipe length and diameters) can be carried out by coupling the graph files generated by our tool and EPANET^31^. Besides, the current clustering algorithm provides a simplified approach based on cost analysis. Further developments of the tool will include meshed network designs, which are common designs to better deal with potential pipe failures, increasing the scope of our tool. Besides, construction costs for the advanced treatments for water reclamation, as well as the operational and maintenance costs for both the water reclamation treatment plant and network, will also be estimated. Moreover, the use of reclaimed water is not accepted in many countries or only accepted for specific purposes according to their regulations. Therefore, an adaptation of our tool based on the country may be considered.

The innovative REWATnet decision support tool to help plan optimal networks for reclaimed water reuse in cities has been developed and tested. With little input data from the users themselves, and by using open data, the tool is able to compute the maximum amount of reclaimed water served per unit of cost invested, including the length and pipe diameters of the network, the location of storage tanks as well as the population served and construction costs. In other words, everything under the same architecture. A comparison of the estimated water consumption for industry/commerce, public and private uses to actual water consumption data gives an overall error of 6.4%. Gaining private user trust in reclaimed water is a key factor for sustainable water reuse networks since these users have the highest consumption rates, 60–70% share of the total in the tested cities (with the differences being due to intense tourism activities). The optimal network graph is computed by using the Mehlhorn routing and clustering algorithms and, when needed, the Limited Budget (LB) availability algorithm. The construction cost of an optimal water reclaimed network for a city of approximately 100,000 inhabitants is estimated to be in the range of €0.17-0.22/m^3^ (for a payback period of 30 years), thus demonstrating a reasonable cost compared to actual costs of drinking water networks. For the same city, the automatic tool computes (in less than 10 minutes) an optimal network able to serve reclaimed water up to three times more than the water served using the current (manual) planning practice. Finally, the tool also provides a map of a user-friendly visualization of the optimal reclaimed water network, including the main and branched networks and colored city clusters, when needed.

## Methods

### Overview

This section first presents the data collection, distinguishing the different data sources; on the one hand, the open data sources to automatically obtain city characteristics and, on the other hand, the consumption and costs-related databases. The combination of multiple data sources is indeed one of the key features of our proposal. Second, the definition of the potential water reuse scenarios is introduced in order to target the desired water destinations in cities based on their water usage. Third, the routing algorithms, based on graph theory and optimization, that support the generation of the reclaimed water network, as well as the algorithms for city clustering (for water tank allocation), pipe diameter selection, and limited budget availability are presented. Finally, the case studies (i.e., cities) used for the testing of the decision support tool are presented.

### City characteristics: open data sources

The city characteristics are collected automatically from open data sources. In particular, we obtain and link together: (i) the city street graph; (ii) the city land plots and building data; and (iii) the city topography.

#### City street graph

The entire city street graph is obtained from the OpenStreetMap^32^ API (a software intermediary that allows two applications to communicate), using the OSMnx library^33^. This API provides a street graph containing the city streets (edges) and intersection points or direction changes on street turns (nodes). It is assumed that the pipes for the new reclaimed water network will be installed following the streets of the city. This information layer is the basis for the data aggregation related to the city (i.e., land plots and building data, water uses, inhabitants, and consumption), and for later running the algorithms.

#### Land plots and building data

The land plots and building data of a city are required to extract all the possible reclaimed water destinations and consumption demands. Each land plot geographical location is linked and clusterized to the nearest points of the city street graph, where the pipes of the reclaimed water network will be placed. The land plot data provides the surface and terrain use (e.g., household, hotels, gardens, or sports facilities), while the building data indicates whether the plot is occupied by a single household or a building of several floors and apartments. The building data is needed to estimate the number of inhabitants per plot, which is required to estimate daily water consumption. The water consumers living in a household are estimated as the ratio between the number of inhabitants in a given city and the number of households of that city, which can usually be obtained from the national statistics institutes.

#### City topography

The city topography is required to know the elevation of the plots, which is essential to calculate the optimal route for the reclaimed water network and place water tanks properly, and assure adequate pipe diameter and minimal operation and maintenance costs due to pumping.

### Consumption and construction costs database

A relational database including the data required to estimate water consumption for different water reuse purposes and network construction investment costs was developed. Default values are included in the database of the decision support tool but all parameters, based on the appearance of new or more precise information available, can be customized to user needs. The database for the estimation of the reclaimed water consumption for different uses is based on bibliographic and practitioner information (see Supplementary Table 4)^34–42^, and can also be extended with additional water uses providing water consumption and destination locations. The database for the reclaimed water network construction costs has been obtained from a tool for life cycle analysis of sewer systems construction^43^, which is based on a standard database frequently used by practitioners^44^ (see Supplementary Table 1, Supplementary Table 2, and Supplementary Table 3).

### Scenario definition

The definition of potential water reuse scenarios involves: (i) the selection of the reclaimed water origin and destinations (among all potential water reuse purposes, see Supplementary Table 4); and (ii) the identification of the city area that will be considered, i.e., the whole city area or only some parts of the city with special interest for water reuse (e.g., new developments) or complying with optional constraints such as a minimum reclaimed water flow rate or population served. The origin of reclaimed water is the centralized wastewater treatment plant, which incorporates a tertiary or advanced treatment for enhancing effluent water quality. The possible uses for reclaimed water would normally be defined by the end user, whereas the areas of application can be either automatically identified by the algorithms or by the user.

### Reclaimed water network generation

In brief, let ${{{\mathcal{G}}}}=({{{\mathcal{V}}}},{{{\mathcal{E}}}})$ be the reclaimed water network graph, with a *V*-element set of nodes ${{{\mathcal{V}}}}$ representing the set of destination (water consumption) nodes, the water source node, and junction points, and an *E*-element set of links ${{{\mathcal{E}}}}\subset {{{{\mathcal{V}}}}}^{ 2 }$ representing pipes. Additionally, *r* (where $r\in {{{\mathcal{V}}}}$) denotes the source node (e.g., the reclaimed water treatment plant or an initial water tank), and ${{{\mathcal{C}}}}$ (where ${{{\mathcal{C}}}}\subseteq {{{\mathcal{V}}}}$) denotes a *C*-element set of consumption nodes. First, we introduce the routing algorithms, and then the optimization algorithms for city clustering (branched network areas definition and water tank allocation), pipe diameter selection, and limited budget availability are presented.

#### Routing algorithms

The routing algorithms to generate and analyze the reclaimed water networks are based on techniques borrowed from graph theory^19^. In the case of water distribution networks, the pipes correspond to the graph edges and the junctions represent the graph nodes from the city street graph. Hence, these networks follow the existing street paths. Using that representation, we generate a network, solving the problem of covering the paths from the centralised water reclamation plant to all the required destinations with minimum costs, using graph routing techniques such as Steiner Tree algorithm variations.

The Steiner tree problem in graphs is well known to be computationally intractable since it is an NP-hard problem^45^. A preliminary performance and complexity study has been carried out on improved Steiner tree optimization and greedy algorithms to select the proper routing algorithm to use. In particular, Table 5 shows the complexity of the Kou^46^, Takahashi^47^, and Mehlhorn^23^ algorithms, and where it can be seen that the Mehlhorn algorithm provides the better complexity.

**Table 5:** Technical comparison of Steiner tree algorithms.

Algorithm	Complexity
Kou	$O(| {{{\mathcal{V}}}}| \times | {{{\mathcal{E}}}}| \times \log | {{{\mathcal{V}}}}| )$
Takahashi	$O\left(| {{{\mathcal{S}}}}| \times | {{{\mathcal{V}}}}{| }^{2}\right)$
Mehlhorn	$O(| {{{\mathcal{V}}}}| \times \log | {{{\mathcal{V}}}}| +| {{{\mathcal{E}}}}| )$

Given ${{{\mathcal{G}}}}=({{{\mathcal{V}}}},{{{\mathcal{E}}}})$, search the Steiner tree with terminals ${{{\mathcal{S}}}}$.

The execution of the routing algorithms over a city street graph results in a reclaimed water network graph ${{{\mathcal{G}}}}=({{{\mathcal{V}}}},{{{\mathcal{E}}}})$, where ${{{\mathcal{E}}}}$ contains the edges of the city street graph that are the most suitable to build the water distribution network (i.e., the pipe route that minimizes the network length). However, in this stage, the pipes defined by ${{{\mathcal{E}}}}$ do not yet contain their diameters.

Since some land plots potentially using reclaimed water may be too far from the nearest node or connection point on the street graph, the routing algorithms omit all plots that are farther away than a certain distance from the land plot centroid, which is given by a threshold in meters customizable by the user.

#### City clustering algorithms

In the case of small cities, a water tank placed alongside the water reclamation plant can be enough to supply all the destination nodes on the distribution network, acting as a unique branched network. Because of large distances, this is not possible in medium to large cities, where a clusterization of the city street network is necessary for scalability reasons. The allocation of additional water tanks along large water reuse networks is needed for practical reasons such as topography issues (i.e., issues that disable gravity distribution), pressure losses, and leak localization^48,49^. Thus, a clustering approach to build the reclaimed water network in medium to large cities is presented.

Although several graph clustering optimization algorithms exist, a medium to large city scenario requires an efficient algorithm to provide a feasible solution in a reasonable amount of time. In Blondel et al. (2008)^22^, the authors propose the so-called Louvain algorithm, a heuristic method based on modularity optimization that outperforms all the other known clustering methods in terms of computation time. Their results show a significant reduction of the network computation time compared to the well-known algorithms of Clauset, Newman and Moore^50^, of Pons and Latapy^51^, and of Wakita and Tsurumi^52^.

Thus, our proposal is to apply the Louvain heuristic algorithm to generate city clusters based on node-pairs proximity, each city cluster representing a branched network area. First, it is necessary to place an initial water tank alongside the distribution network source node (i.e., the water reclamation plant). Next, for each cluster, a simple algorithm optimizes the placement of a water tank. This algorithm selects as candidates the subset of the cluster nodes equal to or higher (in elevation) than the highest destination node. From these candidates, the algorithm finds the node that minimizes the cluster minimum Steiner tree (i.e., that minimizes the branched network area). With this method, we assume that, for each cluster, the water will reach all the destination nodes by gravity. Exceptionally, in order to save costs, the initial water tank also behaves as its cluster water tank. Once the clusters are defined and the water tanks allocated, the main network is constructed based on the minimum Steiner tree between the initial water tank and the other cluster water tanks.

#### Pipe diameter selection algorithm

Once the network graph ${{{\mathcal{G}}}}$ is generated by applying a routing algorithm, it is necessary to select the appropriate construction pipe diameters for each edge from a limited set of available pipe diameters based on the reclaimed water demand of the destination nodes. The diameter selection (DS) algorithm (Algorithm 1) selects the proper pipe diameter for each edge of the reuse water network ${{{\mathcal{G}}}}$. First, the algorithm obtains the expected daily reclaimed water flow volume *w* (in m^3^/s) of each edge $e\in {{{\mathcal{E}}}}$ based on the consumption of the destination nodes $c\in {{{\mathcal{C}}}}$ where the edge *e* is present in the route ${{{\mathcal{E}}}}(r,c)$, $r,r\in {{{\mathcal{V}}}}$ being the water distribution source node. Then, the minimum required diameter *d*(*e*) is computed from the edges expected reclaimed water flow *w*(*e*) and the desired flow speed *s* using the Eq. (1). The flow speed *s* is set to 1 m/s by default, extracted from Simpson and Elhay (2008)^53^, who proposed pipe velocity range of 0.5 to 1.5 m/s. Finally, based on the user-specified set of available pipe diameters ${{{\mathcal{D}}}}$, the algorithm selects for each edge *e* the next greater value ${d}^{{\prime} }(e)$ from the computed minimum required diameter *d*(*e*). Table 6 specifies the full notation used for the diameter selection (DS) algorithm.

1 $$d(e):= \sqrt{\frac{w(e)}{s\times \pi }}\times 2$$

**Table 6:** Full notation concerning the algorithms.

$r,r\in {{{\mathcal{V}}}}$	reclaimed water source node
${{{\mathcal{C}}}}$	set of water distribution consumption nodes; ${{{\mathcal{C}}}}\subseteq {{{\mathcal{V}}}}$
${{{\mathcal{D}}}}$	set of available pipe diameters (each one in mm)
*s*	float constant indicating the desired water flow speed (in m/s, 1 by default)
$m(c),c\in {{{\mathcal{C}}}}$	integer indicating the consumption of destination node *c* (volume, in m^3^)
${{{\mathcal{E}}}}(a,c),c\in {{{\mathcal{C}}}}$	set of edges forming the shortest path from the source node *a* to the destination node *c*; ${{{\mathcal{E}}}}(a,c)\subseteq {{{\mathcal{E}}}}$
$l(e),e\in {{{\mathcal{E}}}}$	float indicating the length the edge *e* (in m)
$w(e),e\in {{{\mathcal{E}}}}$	float indicating the water flow of the edge *e* (in m^3^/s)
${{{\mathcal{X}}}}$	set of edges with assigned water flows; ${{{\mathcal{X}}}}:=\{e:\,e\in f\}$
${{{\mathcal{Y}}}}$	set of edges with unassigned water flows; ${{{\mathcal{Y}}}}:={{{\mathcal{E}}}}\setminus {{{\mathcal{X}}}}$
$d(e),e\in {{{\mathcal{E}}}}$	integer indicating the minimum required diameter of the edge *e* (in mm)
${d}^{{\prime} }(e),e\in {{{\mathcal{E}}}}$	integer indicating the assigned diameter of the edge *e*; ${d}^{{\prime} }(e)\in {{{\mathcal{D}}}}$ (in mm)

##### Algorithm 1

Diameter selection (DS) algorithm.

**Step 1:** Initialize the node *r* and sets ${{{\mathcal{C}}}}$; ${{{\mathcal{D}}}}$; *m*; ${{{\mathcal{E}}}}(r,c),c\in C$; ${{{\mathcal{X}}}}:= \varnothing$; ${{{\mathcal{Y}}}}:= {{{\mathcal{E}}}}$.

**Step 2:** Choose at random an edge with unassigned water flow, i.e., an edge $e\in {{{\mathcal{Y}}}}$, set *w*(*e*) ≔ 0, and update sets ${{{\mathcal{X}}}};{{{\mathcal{Y}}}}$.

**Step 3:** For each water distribution consumption node *c* ∈ *C*:

(a) if $e\in {{{\mathcal{E}}}}(r,c)$, then set *w*(*e*) ≔ *w*(*e*) + *w*(*c*).

**Step 4:** If *w*(*e*) > 0, then:

(a) compute $d(e):= \sqrt{\frac{w(e)}{s\times \pi }}\times 2$, and set ${d}^{{\prime} }(e):= \max ({{{\mathcal{D}}}})$.

(b) for each available pipe diameter $p\in {{{\mathcal{D}}}}$:

(i) if *p* > = *d*(*e*) and $p \,<\, {d}^{{\prime} }(e)$, then set ${d}^{{\prime} }(e):= p$.

**Step 5:** If ${{{\mathcal{Y}}}}\,\ne \,\varnothing$, then go to Step 2.

**Step 6:** If ${{{\mathcal{Y}}}}=\varnothing$, then stop (${d}^{{\prime} }(e)$ contains the assigned pipe diameter $\forall e\in {{{\mathcal{E}}}}$).

#### Limited budget availability algorithm

The limited budget availability (LB) algorithm (Algorithm 2) uses the routing algorithms and the DS algorithm (Algorithm 1) to build a reclaimed water network that maximizes the water volume served for a specific budget *B*. The LB algorithm follows a greedy approach that is an adaption of the algorithm provided in^54^, which presents fast heuristics for the Steiner tree problem with revenues, budget, and hop constraints. The main idea of the algorithm is to iteratively build a reclaimed water network while its cost does not exceed the provided budget. It starts from an initial graph ${{{\mathcal{T}}}}$ with only the reclaimed water source node *r*. For each iteration, and while the construction costs are below the budget, the algorithm adds to ${{{\mathcal{T}}}}$ the destination node *c* ($c\,\notin\, {{{\mathcal{T}}}}$) that provides the best profit *P* (water served per cost ratio). The profit *P* is obtained by dividing the node *c* cubed daily water consumption by the extra construction cost of adding *c* to the graph ${{{\mathcal{T}}}}$.

##### Algorithm 2

Limited Budget availability (LB) algorithm.

**Step 1:** Initialize the node *r*, the budget *B*, and sets ${{{\mathcal{C}}}}$; ${{{\mathcal{D}}}}$; *m*.

**Step 2:** Let ${{{\mathcal{T}}}}$ be the initial graph with ${{{{\mathcal{V}}}}}^{{\prime} }:= \{r\}$ and ${{{{\mathcal{E}}}}}^{{\prime} }:= \varnothing$.

**Step 3:** Set the profit *P* ≔ 0, the iteration candidate node $n:= \varnothing$, and its current network’s closest node $o:= \varnothing$.

**Step 4:** For each reclaimed water consumption node $c:c\in {{{\mathcal{C}}}},c\,\notin\, {{{{\mathcal{V}}}}}^{{\prime} }$:

(a) Get the node $a\in {{{{\mathcal{V}}}}}^{{\prime} }$ that minimizes the path to join ${{{\mathcal{T}}}}$ with *c*, such that:

$$\sum l(e),e\in {{{\mathcal{E}}}}(a,c):= \min ((\sum l(e),e\in {{{\mathcal{E}}}}({v}^{{\prime} },c)),{v}^{{\prime} }\in {{{{\mathcal{V}}}}}^{{\prime} })$$

(b) Copy the graph ${{{\mathcal{T}}}}$ to ${{{\mathcal{U}}}}$, such that $({{{{\mathcal{V}}}}}^{{\prime\prime} },{{{{\mathcal{E}}}}}^{{\prime\prime} }):= ({{{{\mathcal{V}}}}}^{{\prime} },{{{{\mathcal{E}}}}}^{{\prime} })$.

(c) Add the (*a*, *c*) path to graph ${{{\mathcal{U}}}}$, such that ${{{{\mathcal{V}}}}}^{{\prime\prime} }:= {{{{\mathcal{V}}}}}^{{\prime\prime} }\bigcup \{a\}$, and ${{{{\mathcal{E}}}}}^{{\prime\prime} }:= {{{{\mathcal{E}}}}}^{{\prime\prime} }\bigcup {{{\mathcal{E}}}}(a,c)$.

(d) Compute Algorithm 1 (DS) with ${{{\mathcal{U}}}}$ and ${{{\mathcal{D}}}}$, to obtain the pipe diameters ${d}^{{\prime} }(e),e\in {{{{\mathcal{E}}}}}^{{\prime\prime} }$.

(e) Calculate the pipe network construction cost *Z* of ${{{\mathcal{U}}}}$ (including the initial water tank) from ${d}^{{\prime} }(e)$ and *l*(*e*), $e\in {{{{\mathcal{E}}}}}^{{\prime\prime} }$ (see Supplementary Table 1 and Supplementary Table 3).

(f) If *Z* < = *B*, then:

(i) Compute the profit ${P}^{{\prime} }$ of adding *a* to ${{{\mathcal{T}}}}$, such that ${P}^{{\prime} }:= \frac{m{(a)}^{3}}{L}$, where $L:= \sum l(e),e\in {{{\mathcal{E}}}}(a,c)$.

(ii) If ${P}^{{\prime} }\, > \,P$, then set $P:= {P}^{{\prime} }$, *n* ≔ *a*, and *o* ≔ *c*.

**Step 5:** If *P* > 0, then:

(a) Add the (*n*, *o*) path to graph ${{{\mathcal{T}}}}$, such that ${{{{\mathcal{V}}}}}^{{\prime} }:= {{{{\mathcal{V}}}}}^{{\prime} }\bigcup \{o\}$, and ${{{{\mathcal{E}}}}}^{{\prime} }:= {{{{\mathcal{E}}}}}^{{\prime} }\bigcup {{{\mathcal{E}}}}(n,o)$.

(b) Go to Step 3.

**Step 6:** ${{{\mathcal{T}}}}$ represents the final reclaimed network graph ${{{\mathcal{G}}}}$.

### Case studies

The usefulness of the decision support tool presented here is illustrated in the cities of Girona and Lloret de Mar, both in Catalonia (North-East of the Iberian Peninsula), two different but complementary cities in terms of size, density and topography. Girona with its 103,369 inhabitants and 47,446 households (2.4 citizen per household), is a typical Western Mediterranean city; compact, with mixed uses and clearly divided between the old town and the modern peripheral. Its urban area extends 12.7 km^2^ on a rivers’ crossing, has a population density of 8139 hab/km^2^, an average slope of 5.1 and an altitude range (difference between minimum and maximum altitudes) of 177 m. Lloret de Mar is a city located on the northeastern Mediterranean coast of Spain. The city has a year-round population of 39,089 and a seasonal population equivalent (non-residents who either reside, work, study or spend holidays in Lloret de Mar multiplied by a weighting factor based on the total number of days in a year the person stays in Lloret de Mar) of 16,305 (leading to 2.35 citizen per household). Its urban area extends 7.8 km^2^, it has a population density of 5011 hab/km^2^, an average slope of 13.3 and an altitude range of 344 m. Much of the city’s economy is dependent on tourism. In fact, the city has about 120 hotels, which translates into 29,147 hotel beds with a year-round average occupancy rate of about 65% in 2016^34^. In addition, the number of visits to the city in 2014 surpassed one million (Lloret Turisme Press Office). Real water consumption data from 2019 was provided by the water public utility of Girona for the validation of the decision support tool^42^.

The scenarios illustrated in the results section of this paper include: (i) a comparison of the reclaimed water networks generated by different routing algorithms for public water uses in the cities of Girona and Lloret de Mar; (ii) with the best routing algorithm, a comparison of the reclaimed water networks generated for scenarios with only public water use and with both public and private water uses; and (iii) the optimal reclaimed water network with limited budget availability for the case of Girona compared with current practice (i.e., semi-manual approach). The results have been obtained using an Ubuntu 20.04 LTS server (CPU AMD Ryzen 5600X, 32GB RAM), although the tool can be used on other systems. All the computations have been spawned in a Python notebook (Jupyter Hub).

## Supplementary information

Supplementary materials

## Data Availability

Datasets and algorithms related to this study will be made available upon request to the corresponding author.

## Code availability

Code implementations related to this study will be made available upon request to the corresponding author.

## References

[1] Agency, E.E. European waters assessment of status and pressures 2018. *EEA Report*, **7** (2018).

[2] Gössling S (2015). New performance indicators for water management in tourism. Tour Manag..

[3] Mendoza E, Ferrero G, March Slokar Y, Amores X, Azzellino A, Buttiglieri G (2022). Water management practices in euro-mediterranean hotels and resorts. Int. J. Water Resour. Dev..

[4] Deyà Tortella B, Tirado D (2011). Hotel water consumption at a seasonal mass tourist destination. The case of the island of Mallorca. J. Environ. Manag..

[5] Masi F, Langergraber G, Santoni M, Istenic D, Atanasova N, Buttiglieri G (2020). Possibilities of nature-based and hybrid decentralized solutions for reclaimed water reuse. Wastewater Treatment and Reuse - Present and Future Perspectives in Technological Developments and Management Issues. Adv. Chem. Pollut. Environ. Manag. Prot..

[6] Chrispim MC, Nolasco MA (2017). Greywater treatment using a moving bed biofilm reactor at a university campus in Brazil. J. Clean. Prod..

[7] Domènech L, Saurí D (2010). Socio-technical transitions in water scarcity contexts: Public acceptance of greywater reuse technologies in the Metropolitan Area of Barcelona. Resour. Conserv. Recycl..

[8] Vallès-Casas M, March H, Saurí D (2016). Decentralized and user-led approaches to rainwater harvesting and greywater recycling: the case of Sant Cugat del Vallès, Barcelona, Spain. Built Environ..

[9] Santana MVE, Cornejo PK, Rodríguez-Roda I, Buttiglieri G, Corominas L (2019). Holistic life cycle assessment of water reuse in a tourist-based community. J. Clean. Prod..

[10] Castillo A (2016). Validation of a decision support tool for wastewater treatment selection. J. Environ. Manag..

[11] Poch M, Comas J, Rodríguez-Roda I, Sànchez-Marrè M, Cortés U (2004). Designing and building real environmental decision support systems. Environ. Model Softw.

[12] Chhipi-Shrestha G, Hewage K, Sadiq R (2017). Fit-for-purpose wastewater treatment: Conceptualization to development of decision support tool (i). Sci. Total Environ..

[13] Sadr SM (2018). A multi expert decision support tool for the evaluation of advanced wastewater treatment trains: A novel approach to improve urban sustainability. Environ. Sci Policy.

[14] Van Afferden M, Cardona JA, Müller RA, Lee MY, Subah A (2015). A new approach to implementing decentralized wastewater treatment concepts. Water Sci. Technol..

[15] Khurelbaatar G, Al Marzuqi B, Van Afferden M, Müller R, Friesen J (2021). Data reduced method for cost comparison of wastewater management scenarios–case study for two settlements in Jordan and Oman. Front. Environ. Sci..

[16] del Teso R, Gómez E, Estruch-Juan E, Cabrera E (2019). Topographic energy management in water distribution systems. Water Resour. Manag..

[17] Khurelbaatar G (2021). Management of urban stormwater at block-level (MUST-B): a new approach for potential analysis of decentralized stormwater management systems. Water.

[18] Yerri S, Piratla KR (2019). Decentralized water reuse planning: Evaluation of life cycle costs and benefits. Resour Conserv Recycl.

[19] Kesavan HK, Chandrashekar M (1972). Graph-theoretic models for pipe network analysis. J. Hydraulics Div..

[20] Ahmadullah R, Dongshik K (2016). Designing of hydraulically balanced water distribution network based on GIS and EPANET. Int. J. Adv. Comput. Sci. Appl.

[21] Calle E (2021). Optimal selection of monitoring sites in cities for sars-cov-2 surveillance in sewage networks. Environ. Int..

[22] Blondel VD, Guillaume J-L, Lambiotte R, Lefebvre E (2008). Fast unfolding of communities in large networks. J. Stat. Mech. Theory Exp..

[23] Mehlhorn K (1988). A faster approximation algorithm for the steiner problem in graphs. Inf. Process Lett..

[24] Instituto Geográfico Nacional: Instituto Geográfico Nacional, Digital Elevation Model provider for Spain. https://www.ign.es (2019).

[25] Pezoa, F., Reutter, J. L., Suarez, F., Ugarte, M. & Vrgoč, D. Foundations of json schema. Proceedings of the 25th International Conference on World Wide Web 263–273 (2016).

[26] Van Rossum, G. & Drake Jr, F.L. Python Reference Manual. Department of CS, CWI, R 9525 (1995).

[27] Olbricht, R. et al. Overpass API. https://dev.overpass-api.de/index.html (2011).

[28] Gillies, S. et al. Shapely: manipulation and analysis of geometric objects. https://github.com/Toblerity/Shapely (2007).

[29] Mind engineers: Informe tècnic d’abastament d’aigua potable gestionat per aigües de Girona, Salt i Sarrià de Ter, S.A. Technical Report 1, Aigües de Girona, Salt i Sarrià de Ter. (2014). https://seu.girona.cat/portal/dades/web/doc/2014_auditoria_AGISSA_InformeTecnic.pdf.

[30] Atanasova N, Dalmau M, Comas J, Poch M, Rodr¡guez-Roda I, Buttiglieri G (2017). Optimized MBR for greywater reuse systems in hotel facilities. J. Environ. Manag..

[31] Rossman, L. A. et al. EPANET 2: users manual.US Environmental Protection Agency. (2000). https://www.epa.gov/water-research/epanet.

[32] OpenStreetMap contributors: City data retrieved from https://planet.osm.org. (2017). https://www.openstreetmap.org.

[33] Boeing G (2017). OSMnx: New methods for acquiring, constructing, analyzing, and visualizing complex street networks. Comput. Environ. Urban Syst..

[34] Catalan Statistics Institute: Girona population. [Online; accessed 21-dec-2020] (2019). https://www.idescat.cat/emex/?id=170792.

[35] Gabarda-Mallorquí A, Garcia X, Ribas A (2017). Mass tourism and water efficiency in the hotel industry: A case study. Int. J. Hosp. Manag..

[36] Garcia Acosta, X. Nous processos d’urbanització i consum d’aigua per a usos domèstics una exploració de relacions a l’àmbit gironí. (2012). http://www.tdx.cat/handle/10803/109220.

[37] Gascon, L., Arregui, F., Cobacho, R. & Cabrera, E. Urban Water Demand in Spanish Cities By Measuring End Uses. 2004 Water Sources Conference 11, (2004).

[38] Salas, J. J. Cuantificación y caracterización de mis aguas residuales. [Online; accessed 27-jul-2021] (2020). https://www.iagua.es/blogs/juan-jose-salas/cuantificacion-y-caracterizacion-mis-aguas-residuales-i.

[39] March JG, Gual M, Orozco F (2004). Experiences on greywater re-use for toilet flushing in a hotel (mallorca island, spain). Desalination.

[40] Ministerio de Fomento Código Técnico de la Edificiación. (2019). https://www.codigotecnico.org/.

[41] Boneta Herrero A, Rufí-Salís M, Ercilla Montserrat M, Gabarrell Durany X, Rieradevall J (2019). Agronomic and environmental assessment of a polyculture rooftop soilless urban home garden in a mediterranean city. Front. Plant Sci..

[42] Girona municipality Quantification and location of water consumption in the city of Girona. Annual Technical report. (2019). https://terra.girona.cat/apps/observatori/media/observatori/estudis/152/fitxers/basics_consumsaigua19_tot.pdf.

[43] Morera S, Remy C, Comas J, Corominas L (2016). Life cycle assessment of construction and renovation of sewer systems using a detailed inventory tool. Int J Life Cycle Assess.

[44] Banc BEDEC: Structured data bank of building elements. Funded by the Spanish Ministry of Economy and Competitivenes (2013). https://itec.cat/nouBedec.c/bedec.aspx.

[45] Garey MR, Graham RL, Johnson DS (1977). The complexity of computing steiner minimal trees. SIAM J. Appl. Math.

[46] Kou L, Markowsky G, Berman L (1981). A fast algorithm for steiner trees. Acta Inform.

[47] Takahashi H, Matsuyama A (1980). An approximate solution for the steiner problem in graphs. Math. Japonic.

[48] Escribá Bonafé, D. Hidráulica para ingenieros. Librería Editorial Bellisco. Madrid (1988).

[49] Aqualia: NORMAS TÉCNICAS DE ABASTECIMIENTO DE AGUA. Aqualia, (2013).

[50] Clauset A, Newman ME, Moore C (2004). Finding community structure in very large networks. Phys. Rev. E.

[51] Pons P, Latapy M (2006). Computing communities in large networks using random walks. J. Graph Algorithms Appl.

[52] Wakita, K. & Tsurumi, T. Finding community structure in mega-scale social networks. Proceedings of the 16th International Conference on World Wide Web 1275-1276 (2007).

[53] Simpson, A. R. & Elhay, S. Formulating the water distribution system equations in terms of head and velocity. Water Distribution Systems Analysis 2008. *Trans. Am. Soc. Civ. Eng.* 1–13 (2008).

[54] Costa AM, Cordeau J-F, Laporte G (2008). Fast heuristics for the steiner tree problem with revenues, budget and hop constraints. Eur. J. Oper. Res..

