# Supplementary material for: Optimal design of water reuse networks in cities through decision support tool development and testing
Source: NPJ Clean Water. 2023 Mar 17;6(1):23. doi: 10.1038/s41545-023-00222-4 (PMC10020772; doi:10.1038/s41545-023-00222-4)
Supplement: Supplementary file 1 — Supplementary materials [file 41545_2023_222_MOESM1_ESM.pdf]

Supplementary materials

Supplementary Figure 1 and Supplementary Figure 2 illustrate the reclaimed water network visualization in the case study of Girona, in particular for scenario (ii) of public and private uses and for three and seven clusters. The reclaimed networks generated by the Limited Budget availability (LB) algorithm from €500 - 2,000K budget are illustrated in Supplementary Figure 3, Supplementary Figure 4, Supplementary Figure 5, Supplementary Figure 6, Supplementary Figure 7, Supplementary Figure 8, and Supplementary Figure 9.

Supplementary Table 1 illustrates the construction unit cost (per meter) of the reclaimed water distribution network as a function of the pipe diameter. The material considered is designation PE 100 polyethylene tubes. Furthermore, since valves are needed for each pipe bifurcation, Supplementary Table 2 shows the cost of the valves for each available diameter. Supplementary Table 3 also specifies the total construction cost of water tanks, including their necessary facilities, such as a small maintenance building.

Supplementary Table 4 details the consumption rate in L/d (liters per day) per household or per m^2 for the different water uses, including: (i) toilet flushing in households, hotels, camps, restaurants, offices, shopping centres, and sports centres; and (ii) irrigation in household gardens, public gardens, football fields, and vegetable gardens.

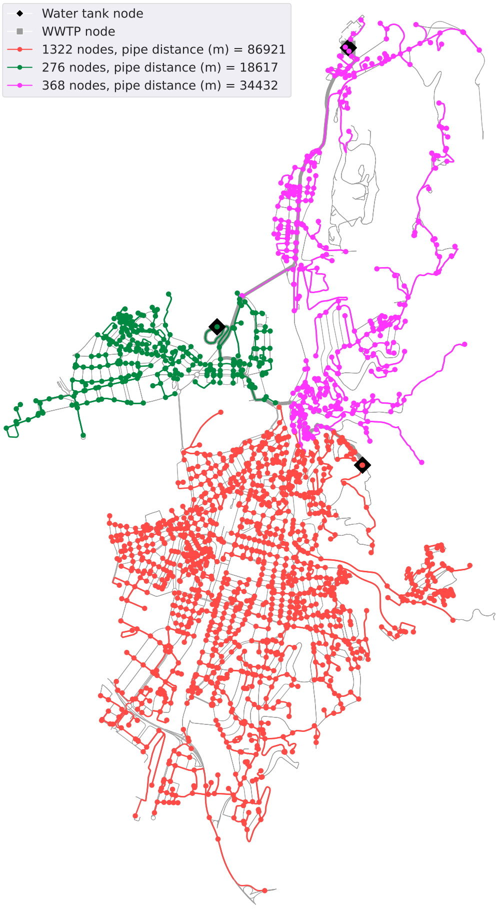

Supplementary Figure 1: Reclaimed water network visualization (I). Visual representation of the reclaimed water network in Girona, considering three clusters and both public and private uses.

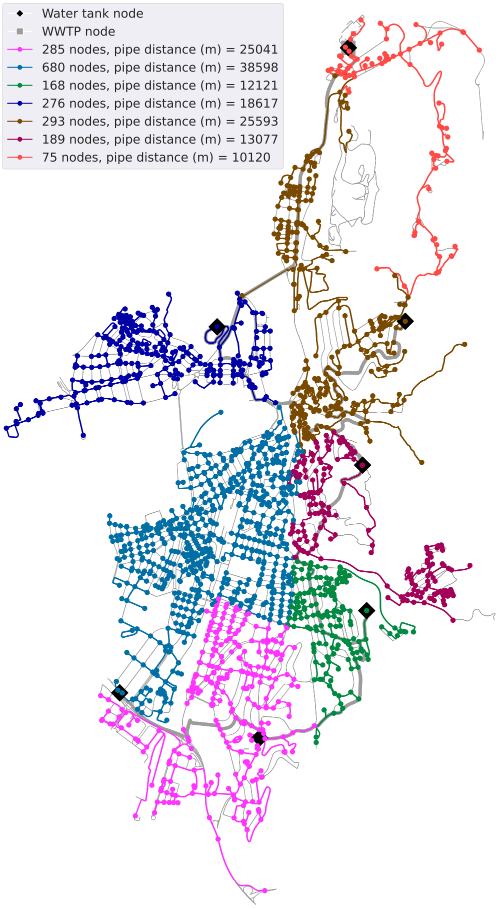

Supplementary Figure 2: Reclaimed water network visualization (II). Visual representation of the reclaimed water network in Girona, considering seven clusters and both public and private uses.

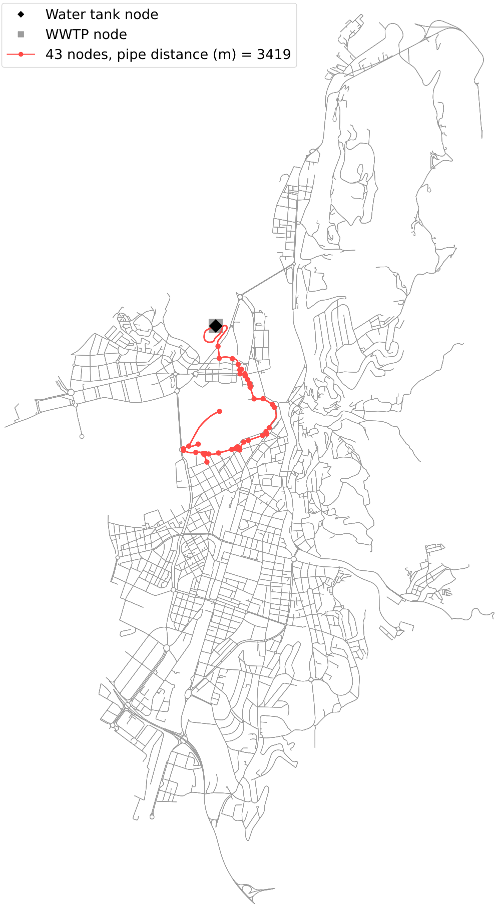

Supplementary Figure 3: Limited budget results (€ 500K). Visual representation of the reclaimed water network in Girona, considering a limited budget of € 500K.

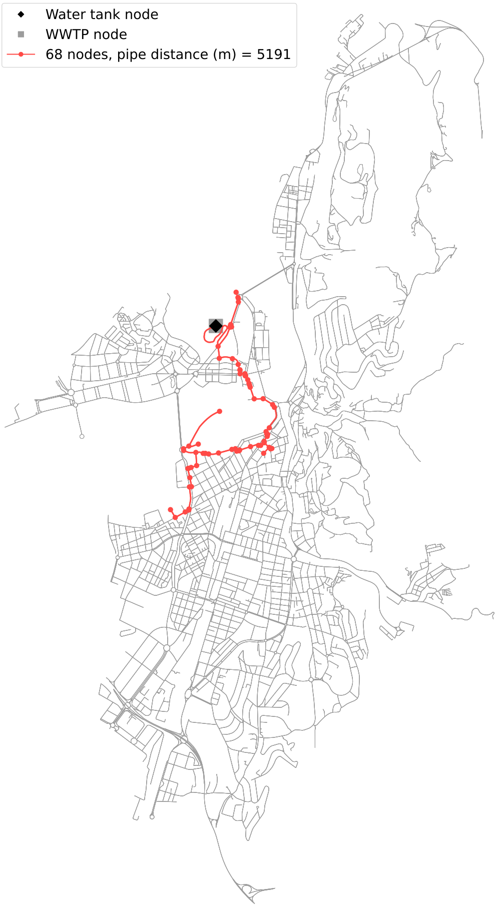

Supplementary Figure 4: Limited budget results (€ 750K). Visual representation of the reclaimed water network in Girona, considering a limited budget of € 750K.

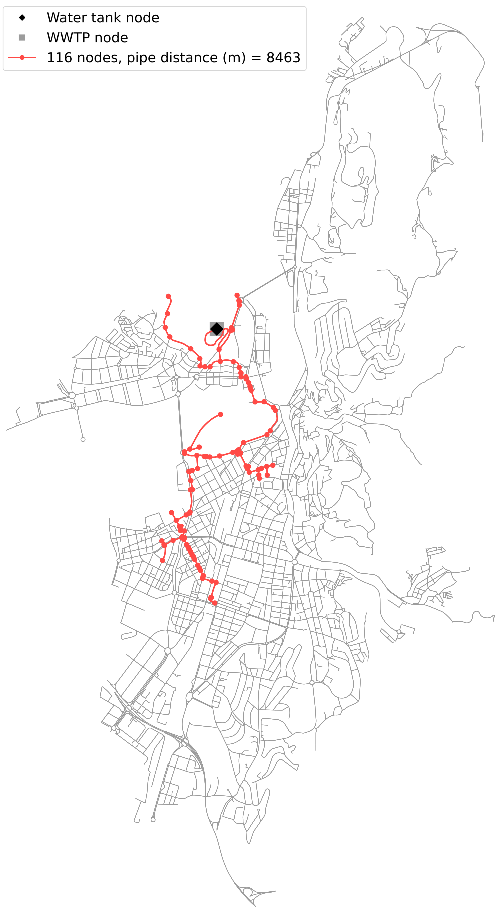

Supplementary Figure 5: Limited budget results (€ 1,000K). Visual representation of the reclaimed water network in Girona, considering a limited budget of € 1,000K.

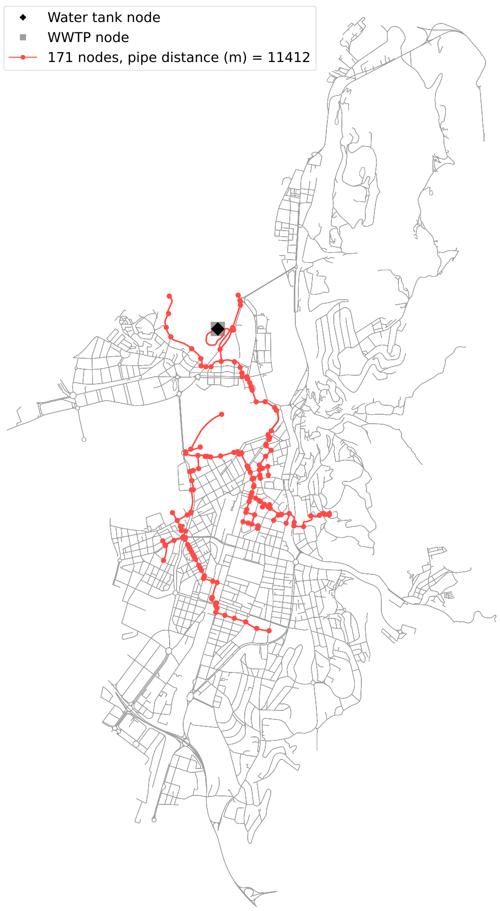

Supplementary Figure 6: Limited budget results (€ 1,250K). Visual representation of the reclaimed water network in Girona, considering a limited budget of € 1,250K.

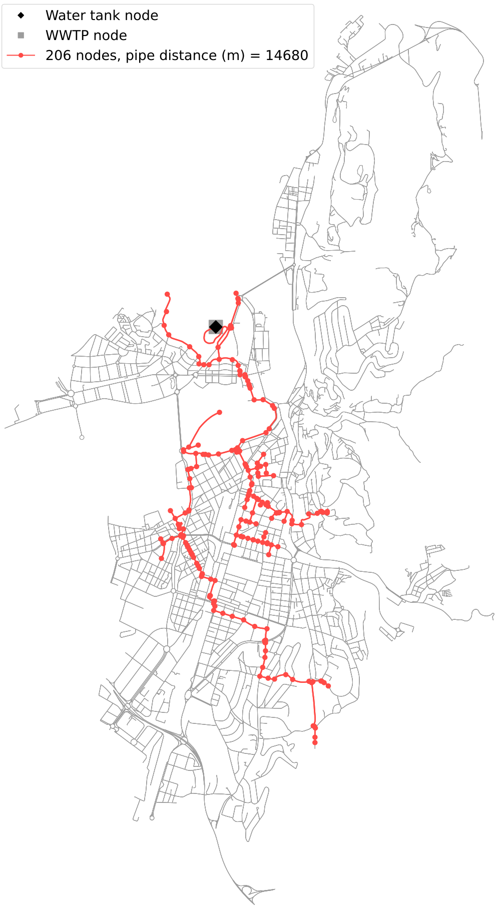

Supplementary Figure 7: Limited budget results (€1,500K). Visual representation of the reclaimed water network in Girona, considering a limited budget of €1,500K.

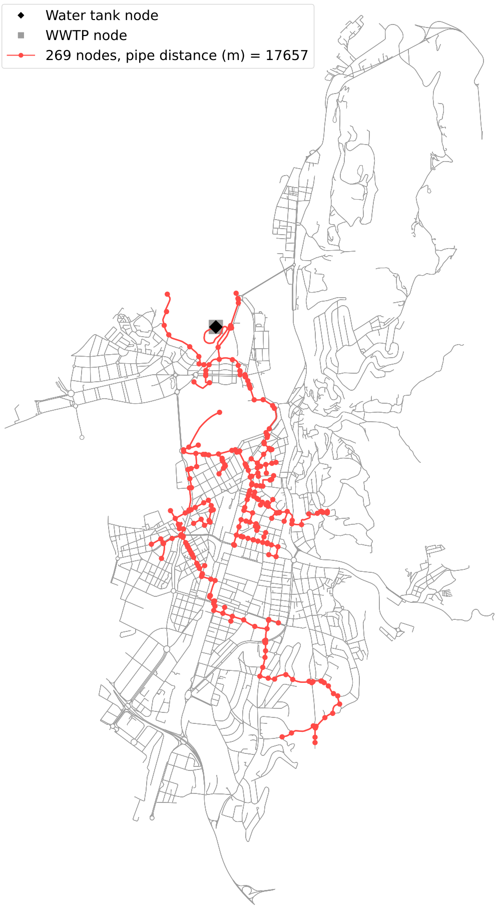

Supplementary Figure 8: Limited budget results (€1,750K). Visual representation of the reclaimed water network in Girona, considering a limited budget of €1,750K.

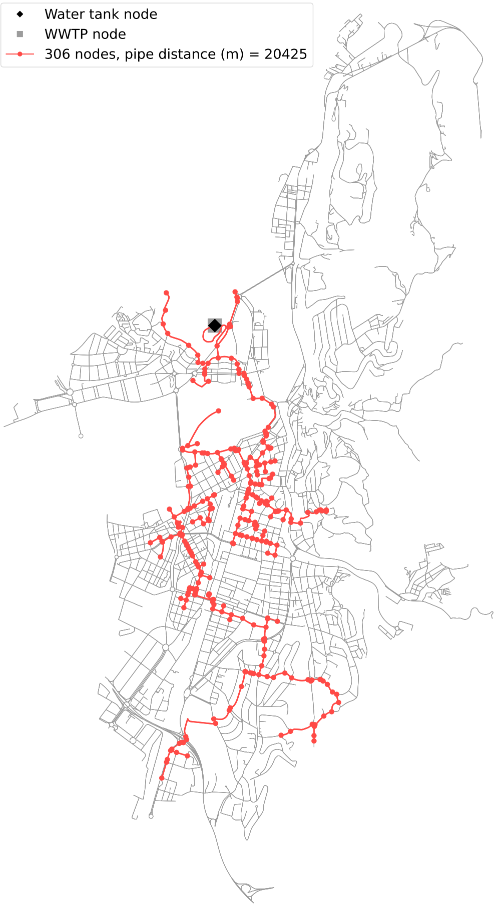

Supplementary Figure 9: Limited budget results (€2,000K). Visual representation of the reclaimed water network in Girona, considering a limited budget of €2,000K.

Supplementary Table 1: Network construction cost per meter.

Pipe (mm)	Material cost (€)	Labour force cost (€)	Total cost (€)	Pipe (mm)	Material cost (€)	Labour force cost (€)	Total cost (€)
63	1.71	69.13	74.38	200	16.11	112.01	134.53
75	2.43	71.33	77.45	225	20.43	125.76	153.50
90	3.49	72.97	80.28	250	25.04	139.50	172.77
110	4.94	74.62	83.54	315	39.83	167.00	217.17
125	6.29	76.82	87.27	400	64.07	194.49	271.49
140	7.92	79.02	91.29	450	112.95	205.77	334.66
160	10.31	81.77	96.68	560	173.57	230.55	424.33
180	13.05	98.27	116.89	630	223.13	242.95	489.38

Material cost (€) – includes the cost of the pipe itself, Labour force cost (€) – includes the cost of the construction workers and the static cost of opening and closing the ditch (€57.03), Total cost (€) – specifies the total construction cost per meter including the material cost, labour force cost and an additional 5% as indirect costs.

Supplementary Table 2: Valve costs.

Valve (mm)	Cost (€)	Valve (mm)	Cost (€)
40	89.29	100	210.88
50	100.46	125	278.35
65	125.77	150	334.97
80	169.88	200	650.00

Valve (mm)	Cost (€)	Valve (mm)	Cost (€)
250	865.55	450	3,095.43
300	1,116.81	500	4,058.26
350	1,812.51	600	8,026.65
400	2,388.50	700	9,014.04

Supplementary Table 3: Water tank costs.

Capacity (m^3)	Cost (€)
400	240,000
2,500	350,000
5,000	440,000
10,000	560,000
20,000	760,000

Supplementary Table 4: Water uses consumption rate.

Water use	Value	Unit	Model	Reference
Toilet flushing household	67.5	L/(d· household)	2.5 hab/household × 6 L/flush × 4.5 flushes/hab·d	Obtained from the statistics of the number of inhabitants per household [34].
Toilet flushing hotel	3.6	$L/(d \cdot m^2)$	0.1 pax/m^2 × 6 L/flush × 6 flushes/pax·d	Obtained from [35-39].
Toilet flushing camp	0.87	$L/(d \cdot m^2)$	0.04 pax/m^2 × 6 L/flush × 3.4 flushes/pax·d	
Toilet flushing restaurant	0.6	$L/(d \cdot m^2)$	0.1 pax/m^2 × 6 L/flush × 1 flushes/pax·d	
Toilet flushing office	2.04	$L/(d \cdot m^2)$	0.1 pax/m^2 × 6 L/flush × 3.4 flushes/pax·d	
Toilet flushing shopping centre	1.2	$L/(d \cdot m^2)$	0.2 pax/m^2 × 6 L/flush × 1 flush/pax·d	
Toilet flushing sports centre	1.2	$L/(d \cdot m^2)$	0.2 pax/m^2 × 6 L/flush × 1 flush/pax·d	
Household garden irrigation	0.49	$L/(d \cdot m^2)$	2 $L/(d \cdot m^2)$ × 90 d / 365 d	Obtained from [40-41].
Football field irrigation	1.48	$L/(d \cdot m^2)$	6 $L/(d \cdot m^2)$ × 90 d / 365 d	
Vegetable garden irrigation	0.99	$L/(d \cdot m^2)$	4 $L/(d \cdot m^2)$ × 90 d / 365 d	
Public garden irrigation	0.42	$L/(d \cdot m^2)$	144,864,000 L / (944,223 m^2 × 365 d)	Obtained from Girona real consumption [42].
